# Psychiatric consultation requests by inpatient medical teams: an observational study

**DOI:** 10.1186/s12913-018-3171-1

**Published:** 2018-05-08

**Authors:** Carla Pezzia, Jacqueline A. Pugh, Holly J. Lanham, Luci K. Leykum

**Affiliations:** 1 Department of Medicine, University of Texas Health Science Center at San Antonio, 7703 Floyd Curl Drive, San Antonio, TX 78229 USA; 2 Department of Human Sciences in the Contemporary World, University of Dallas, 1845 East Northgate Drive, Irving, TX 75062 USA; 3 South Texas Veterans Health Care System, 7400 Merton Minter, San Antonio, TX 78229 USA; 4 McCombs School of Business, University of Texas At Austin, 2110 Speedway, Austin, TX 78705 USA

**Keywords:** Comorbidity, Inpatient medicine, Psychiatric consultation

## Abstract

**Background:**

We describe the way psychiatric issues are addressed by inpatient medical teams through analysis of discussions of patients with behavioral health concerns and examination of teams’ subsequent consultation practices.

**Methods:**

We observed morning rounds for nine inpatient medical teams for approximately month-long periods, for a total of 1941 observations. We compared discussions of patients admitted for behavioral health related medical conditions between those who did and did not receive a psychiatric consultation, developing categories to describe factors influencing consultation or other management.

**Results:**

Out of 536 patients, 40 (7.5%) received a psychiatry consult. Evaluation of a known concern (i.e., substance use, affective disorder, or suicidal ideation) was the most common reason for referral (41.7%). Requests for medication review were second (30.6%). Thirty patients with concomitant behavioral and medical health issues did not receive a psychiatry consult. Cirrhosis with active substance use was the most common medical diagnosis (15), followed by alcohol withdrawal (9).

**Conclusions:**

Four primary themes emerged from our data: positive identification of behavioral health issues by physicians, medication management as a primary reason for referral, patient preference in physician decision-making, and poor management of substance abuse. Our results identify two potential areas where skills-building for inpatient physicians could have a positive impact: management of medication and of substance abuse management.

## Background

An extensive body of literature indicates high prevalence of psychiatric and behavioral health disorders in hospitalized medical patients [1, 2]. Addressing psychiatric or behavioral issues during admission is clinically important, as it is associated with reduced length of stay, [3–6] decreased hospital resource expenditures, [4, 7] and fewer symptoms of depression [2]. While the benefits of addressing these issues are well documented, they are not consistently addressed during hospitalization [8]. Previous literature suggested a lack of recognition of inpatients’ behavioral health issues by non-psychiatrist physicians and, thus, a lack of appropriate psychiatric consultation as a root cause for unaddressed psychiatric issues. [9, 10] However, more recent research suggests that inpatient physicians do in fact evaluate psychosocial and behavioral health factors alongside acute medical or surgical complaints [8].

Psychiatric and behavioral health consultation rates have remained consistently low over time and previous research may not accurately assess the extent to which inpatient physicians recognize and address patients’ psychiatric conditions directly [2, 11]. Liaison psychiatry and other behavioral health inpatient consulting services are now commonplace, suggesting that availability of services may not be a contributor to low referral rates [1]. Current research continues to focus on the misclassification of psychiatric conditions in hospital patients as indication to why patients are not referred to appropriate services [10]. Few studies have examined the reasons for low rates of consultation with psychiatry services from the perspective of the inpatient physician [12].

The limited available research describing inpatient psychiatry referral practices demonstrates several general trends. Chart review studies suggest associations between physician referral to psychiatry and a patient’s gender, age, previous psychotropic medication use, and documented history of behavioral health issues [9, 13]. It is unclear whether patients who are deemed “management problems” for demanding behavior are more likely to receive an inpatient psychiatric consult [9, 14, 15]. Because referrals to inpatient psychiatric services remains low, and because the best models for organizing inpatient collaborative behavioral health services have not been clearly established [1, 4, 16] updated research on reasons for or against referral from the perspective of inpatient physicians is necessary to address these gaps in the literature and in practice. Automatic referrals to consulting services for every patient with a psychiatric history may not be clinically useful or cost-effective. Thus, it would be helpful to further understand factors that may influence physician decision-making regarding consultation to better deliver inpatient care to patients with comorbid physical and behavioral health conditions.

To the best of our knowledge, there are no published studies that qualitatively examine referral practices from the perspective of the referrer. [12] The purpose of this study is to describe how behavioral health/psychiatric issues are addressed by inpatient medical teams through qualitative analysis of team discussions of patients with behavioral health concerns and examination of teams’ subsequent consultation practices. We were interested in the rate of consultation within the context of understanding the overall approach through which inpatient medical teams might address the behavioral health needs of their patients outside of psychiatric consultation. This information, in turn, may guide the development of future interventions targeting medical and psychiatric services to better serve the complex needs of acutely ill medical patients with behavioral health disorders.

## Methods

As part of a larger project studying physician teams [17, 18], we conducted an observational study of nine inpatient medicine physician teams in two teaching hospitals in an urban setting from 2008 to 2013. Each team was observed daily for four-week periods, with field notes and audio recordings taken. This observational approach in which we followed the physician teams allowed us to see “natural” day to day developments informing patient care, minimizing the potential for recall bias that could occur in retrospective interviews, and allowing us to capture discussions regarding care options that may not be well documented in daily notes.

One hospital in our study is a 614-bed county hospital / Level 1 trauma center for Bexar County in San Antonio, Texas. The second is the 220-bed Veterans Affairs acute care facility for the South Texas Veterans Health Care System. Physicians at both hospitals have consulting access to relevant inpatient services, such as psychiatry and social work. The county hospital also has an additional Licensed Chemical Dependency Counselor (LCDC) inpatient service. The medical teams consisted of a faculty attending physician, one post-graduate year (PGY)-2 or PGY-3 resident, and two PGY-1 interns.

A member of the research team observed morning rounds as a passive observer. Rounds are considered a key time for medical teams to discuss each patient and develop diagnostic and therapeutic plans. Physician presentations typically follow a standard format to include admissions data, continuing care, new concerns, and plans for disposition for each patient. The need for and status of consultations is an expected point of discussion for each presentation. As part of our overall study, we noted all consultations requested for all patients, including specifying the specific services called. During our data collection period, a total of 1941 patient care discussions across 562 patients were observed.

We also collected general information on the patient’s admission, including reason for admission, discharge diagnosis, chronic medical issues and behavioral health / psychiatric history. Behavioral health issues included active substance abuse, affective disorders, and cognitive disorders. Substance abuse included both alcohol and drug use; affective disorders included depression, anxiety or bipolar disorder; and cognitive disorder included schizophrenia, schizoaffective states, or the presence of psychosis with an affective disorder. We did not categorize dementia as a behavioral health issue for our analysis because medical teams at our research sites regularly assess and address dementia as part of the medical treatment plan. However, our identification of consults would have caught any cases of dementia for which psychiatry may have been called. We identified behavioral health issues by their presence in at least one of the following: patient problem lists, admission notes, discharge summaries, or recorded team discussions.

For this analysis, we identified the overall set of patients who received psychiatric consultation, along with the set of patients with active behavioral health or psychiatric issues at the time of admission. To identify this second group, we only included patients for whom a behavioral health or psychiatric issue was an admitting symptom or was listed as an active medical problem in the discharge summary in this analysis, as it was possible that patients with only a history of a psychiatric condition would not have had an active issue that needed to be addressed during the hospitalization. We also included patients admitted with complications of cirrhosis who continued to drink alcohol, as their behavioral health practices clearly had a continued impact on their medical presentation.

To examine reasons why inpatient behavioral health and psychiatric consultations were or were not obtained, we compared discussions about a patient’s behavioral health condition between patients who did receive a psychiatry consultation, and those who had an acute psychiatric or behavioral health issue who did not receive a psychiatric or behavioral health consultation, developing categories to describe influencing factors. For each patient every day, we noted when recommendations from consulting services were discussed by the team. When team rounds included speaking with patients in their rooms, we observed physician-patient interactions and whether the medical team addressed behavioral health concerns directly with the patient.

To conduct an in-depth qualitative analysis of discussions of behavioral health / psychiatric issues and reasons for consultation, we reviewed discussions of all patients who received a psychiatry consult (*n* = 40), and discussions of ten patients with active behavioral or psychiatric issues but who did not receive a psychiatry consult. We selected ten patients to balance the need to have a representative sample with the desire to conduct an in-depth analysis of the daily discussions occurring for each day that patients were hospitalized. We decided to include any patients who had suicidal ideation who did not have a consult, given the potential gravity of the clinical situation. We also decided to sample at least one patient per team to ensure that we captured a broad range of discussions across multiple physicians, and not just the practice pattern of a small group of physicians. Beyond these considerations, patients were randomly chosen.

Physician discussions of the included patients were identified by reviewing the field notes and the audio recordings. For patients who received a psychiatry consult, we focused on analyzing the discussions during which the team decided to call the consult to understand why the referral was made. If the reason for referral was unclear from the discussion on rounds, we obtained the reason from the consult request note in the patient’s chart. For patients for whom a consult was not requested, we reviewed the audio recordings of all discussions for every day that the patient was hospitalized to ensure that we identified any conversations that occurred regarding the patient’s behavioral health or psychiatric condition.

The recordings of patient discussions were transcribed by an external service and checked for accuracy by a research team member. For patients who received a psychiatry referral, transcripts were reviewed for stated reasons for referral. For patients who did not receive a consult, transcripts were reviewed and notes made on any discussion of psychiatric or behavioral health factors relevant to the patient’s condition or hospital stay, any consideration for or against a psychiatry consult, and any attempts by the medical team to address behavioral health concerns in other ways (e.g., discussing with the patient directly or discussing with other services, such as social work). For all patients, transcripts were analyzed using a deductive coding schema. The notes and codes from the transcripts for patients who did and did not receive consultation were then compared for any commonalities that could be categorized as emergent themes and points for discussion. Specifically, we applied codes from the literature on management of behavioral health issues to our notes of discussions of those issues by the teams. For example, we applied the codes of “motivational” and “authoritative” to instances of physician advice-giving, and the code “management problems” to notes regarding referral outcomes. The absence of codes for some patients was also taken into consideration. For example, some cirrhosis patients as noted below completely lacked codes for both advice-giving and management problems.

This study was approved by the Institutional Review Board at the University of Texas Health Science Center at San Antonio, the Research and Development Committee for the South Texas Veterans Health Care System and the Research Committee at University Health System.

## Results

### Rounds characteristics

Rounding styles varied from sitting in the team room discussing patients then seeing some or all patients afterwards to discussing patients in the hall right before seeing them or directly at bedside. Typically, presenters mentioned all items on the “problem list” on a regular basis. However, on some days (e.g., on-call days or when an intern had clinic) the attending would elect to discuss only the most pressing problems and changes in status on other problems. As such, behavioral health conditions as noted on the “problem list” were not always discussed unless it was considered a primary contributing factor to their hospital stay.

### Patient characteristics

**Fig. 1:**
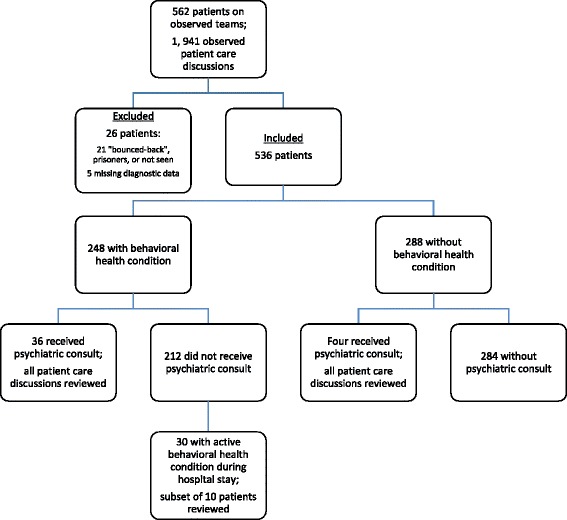
Flow diagram of inclusion criteria and stratification of study sample

Of the 562 patients in our sample, we excluded twenty-one patients who “bounced-back” to another team and did not receive any additional care from the teams we observed. We also excluded incarcerated patients. We were unable to confirm behavioral health and diagnosis data on five patients. Figure 1 shows the progression to our final sample of 536 patients. Table 1 shows general characteristics of this sample. Of this group of 536 patients, 248 (46.3%) had a co-occurring behavioral health condition noted in their medical record. Of those 248 patients with behavioral health conditions, 104 (41.9%) patients had an issue with substance abuse, 184 (74.2%) with affective disorders, and 23 (9.3%) with cognitive disorders. The remaining 289 patients did not have a documented history of any behavioral health condition. The most prevalent medical diagnoses for the two groups of patients were Cirrhosis (9.3%), Cancer (8.1%), and Pneumonia (6.9%) for those with co-occurring behavioral health conditions and Cancer (12.2%), Pneumonia (9.4%), and Diabetes (5.9%) for those without. Overall, 7.5% of the total sample received a psychiatry consult. Of the 248 patients with a behavioral health condition, 14.5% received a psychiatry consult, while of the 289 without a history of behavioral health conditions, only four (1.4%) received a psychiatry consult.

**Table 1:** Patient sample characteristics

	History of mental health issue	No history of mental health issue
Patient sample	248	289
LOS	5.39 days	4.85 days
ULOS	0.6 days	0.3 days
Readmission rate	14.1%	12.8%
Mental health consultation	36	4

### Review of patients who received psychiatry consultation

The four patients without a history of behavioral health conditions were referred to psychiatry for evaluation of newly recognized depression symptoms (1), suicidal ideation (1), and assessment of competency to make medical decisions (2). The remaining 36 received consults for a variety of reasons (Table 2). Evaluation of a known behavioral health concern (i.e., substance use, affective disorder, or suicidal ideation) was the most common reason for referral (41.7%). Requests for medication review were second (30.6%). In these cases, the medical team sought to ensure that outpatient medication type and dosing was appropriate to manage the behavioral health condition, particularly when new inpatient medications were being added. In our sample, physicians never discussed consulting psychiatry for “management problems,” as defined by Steinberg et al. [15]. Indeed, in one clear case of an “oppositional” patient who voiced her distrust of all physicians, the medical team did not seek a psychiatry consult. The physicians respected the patient’s preference and did not request consultation against her wishes. Additionally, three patients requested to speak with someone from psychiatry, and the medical team respected their decision to consult psychiatry in the management of their care.

**Table 2:** Reasons for psychiatric referral

Reasons for Referral	
Mental health condition • Substance use (2) • Affective disorder (6) • Suicidal ideation (7)	15 (41.7%)
Medication Review	11 (30.6%)
Capacity for Medical Decision-Making	2 (5.6%)
Acute Altered Mental Status	1 (2.8%)
Transfer to Psychiatry Service	1 (2.8%)
Transfer to Drug Rehabilitation Program	1 (2.8%)
Requested by Patient	2 (5.6%)
Reschedule of Outpatient Appointment	1 (2.8%)
Multiple Reasons • transfer to drug rehabilitation program, requested by patient, capacity for medical decision-making (1) • medication review, transfer to psychiatry service (1)	2 (5.6%)
Total	36

### In-depth examination of patients without a psychiatry consult

Thirty patients had explicit comorbid psychiatric and medical health issues as noted in their admitting symptoms or reason for admission, but did not receive a psychiatry consult. Cirrhosis with active substance use was the most common medical diagnosis pertaining to a behavioral health condition (15), followed by alcohol withdrawal (9), suicidal ideation (3), affective disorders (2), and cocaine abuse (1). Interestingly, not a single patient with cirrhosis and active alcohol use received a consult to psychiatry or LCDC.

Of the subset of 10 of these patients for whom we reviewed all patient care discussions, two patients were considered too disoriented for referral and one patient’s behavioral health issues (substance abuse, affect disorder, and cognitive disorder) were considered chronic and being managed by outpatient services. The remaining seven patients reviewed were admitted for cirrhosis (5) or alcohol withdrawal (2). Table 3 provides a summary for the team discussions on each of these patients.

**Table 3:** Discussion summaries for patients with active behavioral health condition but did not receive consult

Patient	Diagnosis	Discussion Summary
1	Cirrhosis (active drinker)	No discussion of alcohol use or any other social issues. Medical discussion focused on hernia, potential need for paracentesis, and leaking of fluid.
2	Cirrhosis (active drinker)	Continued alcohol use briefly mentioned: “He says he’s stopped drinking but…” No further discussion.
3	Cirrhosis (active drinker)	Attending engaged patient utilizing motivational interviewing techniques (e.g., “what do you like/dislike about your drinking?”). Patient agreed to speak with Social Work services. Team referred patient to Social Work to address multiple social issues besides alcohol use, including housing and transportation.
4	Passive suicidal ideation	Team discussed mental health issues as chronic and considered suicidal ideation as passive. Psychiatric issues already being followed as outpatient.
5	Cirrhosis (active drinker)	Continued alcohol use discussed directly but not actionably addressed: “he stopped drinking when he started feeling poorly… it’s the culture of alcoholism… he’ll just come back after a few drinks.”
6	Cirrhosis (active drinker)	Continued alcohol use was discussed in relation to his follow-up medical care but not actionably addressed: -“He still drinks.” -“He’s a smart man… He’s an alcoholic, too. Nothing against him, but if he goes home and starts drinking, he’ll miss his appointments.”
7	Alcohol withdrawal	Alcohol use was discussed in relation to symptoms of withdrawal: “sounds like he was in withdrawal when he came in, and he’s about to not be drunk.” No other discussions.
8	Alcohol withdrawal	Team suggested referral to patient. Patient refused both psychiatry and LCDC services. Team physician told patient: “you’ve got to stop the drinking. It’s going to kill you.”
9	CVA, suicidal ideation	Patient too disoriented due to CVA.
10	Gangrene, PTSD	Psychiatry consult was discussed for a capacity evaluation. Patient waiting to be discharged. The discharge physician suggested to intern: “you can make a judgement on capacity.”

The first patient with cirrhosis reviewed did not include any discussion of alcohol use or social issues. In four cases, the patient’s alcohol consumption was discussed amongst the team and recognized to be a contributing factor to his medical condition, but it was not addressed with the patient either by the team or a consulting service. For example, in the case of another patient with cirrhosis, after several diagnostic exams the team considered continued alcohol consumption to be the underlying reason for the current hospital admission. They did not discuss pursuing an intervention to address this issue. Instead, they referred to the “culture of alcoholism” and commented “he’ll just come back after a few drinks.” In two cases, the team directly addressed the alcohol use with the patient. One used authoritative advice giving (“You’ve got to stop the drinking. It’s going to kill you.”), which was typically how we observed the teams interacting with patients regarding their substance abuse issues. The second team’s attending engaged the patient using motivational interviewing techniques. The former patient refused any further LCDC/psychiatry or Social Work services, while the latter accepted the recommendation for Social Work.

## Discussion

In this paper, we explored factors that contributed to how medical teams provide care for hospital patients with psychiatric comorbidities, focusing on decisions to engage psychiatric services consultation. Several themes emerged from our results that are relevant to the literature: recognition of behavioral health issues, medication management, patient preference, and management of substance abuse. First, our study is consistent with previous research indicating that inpatient medical teams recognize behavioral health issues in their patients. [8] Behavioral health conditions listed in the written notes, including admission and discharge summaries, and problem lists of the electronic medical record at each research site indicated that the physician teams acknowledged the patient’s condition, even if it was not directly influencing the current medical condition. Nearly 47% of our patient population was identified as having underlying behavioral health issues, a number that is comparable to studies that actively assess prevalence of psychiatric conditions in medical inpatients [11, 13].

While other previous research highlights the lack of recognition of comorbid behavioral health issues and indicate the need for medical physicians to be better trained, our findings suggest that medical teams do in fact recognize behavioral health issues yet do not consistently use psychiatric consultation services. This finding argues against provider training in recognition of behavioral health disorders as a sole approach for better addressing these issues during hospitalization for acute medical illnesses. We do not mean to suggest that physicians would not benefit from training, but training alone seems unlikely to lead to increased referral rates since psychiatric / behavioral health issues are recognized.

We focused on consultation as a metric for assessing inpatient medical teams’ management of behavioral health issues. However, we do not mean to suggest that all patients with a recognized behavioral health condition should have a psychiatric consultation while in the hospital. With such a high prevalence, the feasibility of having consultation for all patients with a chronic behavioral or psychiatric condition would be limited. Additionally, it is not clear that psychiatric referral makes a significant difference in the hospital experience and outcomes of patients with a behavioral health condition, or whether other approaches to management might be effective. Regardless of the approach to consultation, the medical team serves an important triage function, regularly discussing behavioral health issues during rounds as part of the “problem list.” Tailoring medical physician trainings to focus on triaging need for referral or other management strategies rather than on recognizing the presence of a behavioral illness may be more practical in providing optimal patient care for the complex medical patient. Informing physicians of available outpatient services to recommend when an inpatient referral is not necessary may also prove useful.

Medication management is a particular concern for complex medical patients, and a key theme in our analysis. Our results indicate that medical teams frequently consult psychiatry to ensure that medication regimens are both safe and effective for their patients with behavioral health issues. This finding further supports Ward and Schwartz’s [19] recommendation that hospitalists be better informed about psychotropic medications and use all available resources to address the behavioral health needs of medical patients. However, there are limitations to what a hospitalist may be able to cover on their own, even with more training in psychotropic medications. While many hospitals provide physicians access to online databases or have automated methods to evaluate medication interactions in the electronic health record, the reported usefulness of these databases is variable [20, 21]. Moreover, if an interaction is found to be a concern, a consult would still likely be helpful for deciding on suitable alternative medications. Another potential way to address concerns regarding medication management is through the creation of integrated teams that include either a psychiatrist or pharmacist to address medication related issues for all patients. Indeed, on at least three different teams a pharmacy student accompanied the team for part of the observation period, and the student provided an invaluable resource to immediately address any medication concerns, both by the team asking directly when in doubt and by the student interjecting when the team overlooked conflicting medications or dosages. The immediacy of the information provided through an integrated team also contributes to shortened length of stay [4].

A third important issue that emerged from our analyses was the role of patient preferences in the physician’s decision to consult psychiatry or other services. Patients who were found to have an acute comorbid condition affecting their medical care and voiced acceptance of speaking with psychiatry received a referral. Moreover, in our sample, two patients received a consultation solely because they requested to speak with psychiatry. It is unknown if the medical team may have eventually considered a psychiatry consultation on their own, but team discussions prior to the patient request did not include mention of psychiatry as a potential consult (neither as inpatient nor as outpatient). When a competent patient voiced opposition to psychiatric consultation, the medical team respected the wishes of the patient to refuse services. This was particularly observed in patients with chronic behavioral health issues and ongoing substance abuse issues. Clearly, it would be ineffective to make a referral for patients who do not wish to see psychiatry. Research on integrated teams that include a psychiatrist suggest that patients may be more willing to speak to someone who is already present [4]. How to meaningfully include psychiatrists or behavioral health providers into inpatient teams while also recognizing that they may be a limited resource will be an important question to address. It may involve pharmacists or other behavioral health professionals such as counselors.

Stigma and legal concerns may impact self-disclosure of substance use, but substance abuse issues are often considered among the easiest of behavioral health disorders to diagnose because standard screening questions are now commonplace in medical history intake [2]. Yet our results suggest that these issues are either not well addressed or completely ignored. On the few occasions when the team addressed the substance abuse directly with the patient, strategies felt to be most effective in engaging patients such as motivational interviewing were not consistently used. Team discussions of the “culture” of substance abuse may reflect a general sense of futility in engaging patients with dependency issues. Recent research in trauma patients with severe chemical dependency has shown that motivational interventions are more effective than previously considered [22]. The number of patients who have cirrhosis and continue to drink alcohol warrants further research examining the effectiveness of motivational interventions in the inpatient medical setting. An integrated team that includes a psychiatrist (when cost effective), social worker, or LCDC could also potentially address active substance abuse issues that may contribute to poor patient outcomes.

In summary, our findings suggest the need to further examine potential strategies for meeting the needs of complex medical-psychiatric hospital patients beyond education in recognition. The teams we observed required assistance with medication management, and effectively engaging patients with substance abuse disorders. It may be that hospitalization could be considered a “teachable moment,” particularly for admissions directly related to cirrhosis or alcohol withdrawal. Indeed, some physicians did use the opportunity to address alcohol use directly with some patients. However, this was not done consistently with all patients or using current evidence-based practices. Physician training in evidence-based practices and/or the use of integrated medical – behavioral health teams are warranted, yet there are considerable barriers to expanding some services. With the increasing shortage of behavioral health professionals, it is necessary to identify efficient ways to address key issues, such as the use of pharmacists or counselors.

### Limitations

This exploratory study focuses on a small team and patient sample. It is limited by the fact that we only included two hospitals and only medical teams. However, our prevalence rates were consistent with the literature. This small sample size also allowed us to use an in-depth discursive analytic approach to better understand how inpatient medical teams approach behavioral health issues. We also did not examine to what extent the medical team may have missed new onset behavioral health conditions that could have benefited from inpatient psychiatry consult. Since the rate of recognition is within the prevalence range of previous studies that purposefully screen patients, we do not believe that a large number of new-onset conditions occurred. Finally, it is possible that discussions with the patient and other consulting services occurred outside of the period we observed the team. However, for other components of this study, we observed teams outside of rounds and saw no evidence for these conversations occurring.

## Conclusion

Our results identify two potential areas for improvement with regard to managing hospitalized patients with concomitant psychiatric or behavioral health issues: medication management and effective engagement with patients for substance abuse issues. A skills-building approach to these issues could be warranted based on the high prevalence of patients with behavioral health comorbidity, particularly with regard to substance abuse, and the frequency with which we observed consultation for medication management. An alternative approach would be the creation of integrated, proactive teams. The high prevalence of behavioral health and psychiatric comorbidity among hospitalized medical patients may make a team approach more efficient and effective. Medical teams that incorporate a psychiatrist into their daily rounding routine have been shown to detect psychosomatic conditions earlier in the hospital stay that if not appropriately addressed at the start could lead to unnecessary stress on healthcare resources. [1] More research is necessary to determine appropriate composition of teams to ensure a high benefit-cost ratio. For patients with complex medication regimens, an integrated team psychiatrist or pharmacist may proactively ensure that dosing is appropriate and will not be counter-effective to new medicines being prescribed by the medical team. For patients with substance abuse issues, an integrated team psychiatrist or substance abuse counselor may be more skilled in providing appropriate evidence-based interventions that might otherwise be refused by the suggestion of a referral. Furthermore, integrated, proactive approaches have the potential to shorten length of stay and be financially beneficial, particularly in the current context of greater emphasis on quality and performance-related reimbursement methodologies. [4, 23, 24]

## Abbreviations

CVA: cerebrovascular accident
LCDC: Licensed Chemical Dependency Counselor
LOS: length of stay
PGY: post-graduate year
PTSD: post-traumatic stress disorder
ULOS: unnecessary length of stay
